# Neural Stem Cell‐Derived Extracellular Vesicles Improve CNS Delivery and Therapeutic Efficacy of Antisense Oligonucleotide in Amyotrophic Lateral Sclerosis

**DOI:** 10.1002/jev2.70370

**Published:** 2026-09-15

**Authors:** Yaochao Zheng, Taylor Jade Ellison, Robert Garrard, Jinghui Gao, Xinning Lai, Lin Lan, Jin Xie, Jie Jiang, Yao Yao

**Affiliations:** ^1^ Regenerative Bioscience Center, Department of Animal and Dairy Science, College of agricultural and environmental sciences University of Georgia Athens Georgia USA; ^2^ Department of Chemistry University of Georgia Athens Georgia USA; ^3^ Department of Cell Biology Emory University Atlanta Georgia USA

**Keywords:** amyotrophic lateral sclerosis, antisense oligonucleotide, drug delivery, extracellular vesicles, neural stem cell

## Abstract

Antisense oligonucleotides (ASOs) hold clinical promise for the treatment of neurodegenerative diseases such as amyotrophic lateral sclerosis (ALS). Yet, their efficacy is constrained by the need for invasive delivery, poor central nervous system (CNS) penetration, inefficient cellular uptake and rapid clearance. Here, we developed neural stem cell‐derived extracellular vesicles (NSC‐EVs) as natural carriers to enhance CNS targeting and therapeutic efficacy of ASO‐based treatments for ALS. Using a passive loading method, therapeutic ASOs were efficiently associated with NSC‐EVs (NSC‐EV‐ASO). Compared with free ASOs, NSC‐EVs‐ ASOs exhibited markedly enhanced neuronal uptake in vitro and increased CNS accumulation and neuronal localization in vivo following intracerebroventricular, intranasal, or intravenous administration. Functionally, NSC‐EV‐ASOs reduced the mutant SOD1 protein expression and the pathogenic hexanucleotide repeats in the *C9orf72* loci in ALS cellular models. In the ALS mouse model, intranasal administration of NSC‐EV‐ASOs more efficiently delayed disease onset, improved motor performance, preserved motor neurons and attenuated astrocyte activation during the study period compared with free ASOs. These findings provide proof‐of‐concept evidence supporting the potential of NSC‐EV as a promising platform that not only enhances CNS delivery of ASOs but also contributes to overall therapeutic activity, offering a synergistic strategy to advance nucleic acid therapeutics for ALS and potentially other neurological diseases.

## Graphical abstract

Passive ASO association with NSC‐EVs enhances neuronal uptake and CNS accumulation. NSC‐EV‐ASOs suppress pathogenic targets in ALS cellular models and, following intranasal administration, delay disease onset and improve motor function in SOD1‐G93A mice. These findings provide proof‐of‐concept evidence supporting NSC‐EVs as a platform for enhancing CNS delivery and therapeutic activity of ASO therapeutics in ALS.

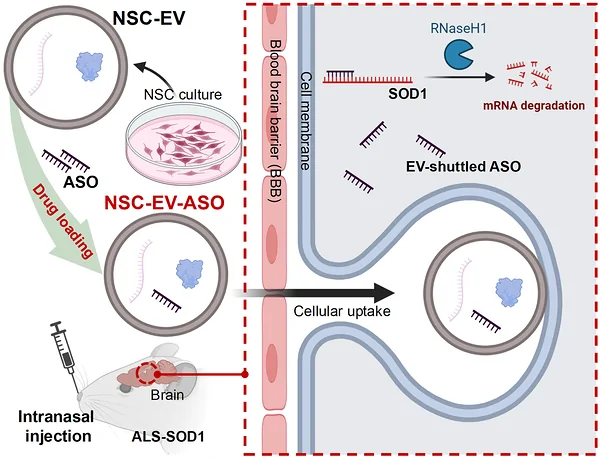

## 1 Introduction

Amyotrophic lateral sclerosis (ALS) is a fatal neurodegenerative disease characterized by progressive degeneration of upper and lower motor neurons, leading to paralysis and death, typically within 3–5 years of symptom onset (Ilieva et al. 2023). Despite advances in understanding its pathogenic mechanisms, effective treatments remain limited. Approximately 10% of ALS cases are familial, with most caused by mutations in *superoxide dismutase 1 (SOD1)* and hexanucleotide repeat expansions (HREs) in *Chromosome 9 open reading frame 72 (C9orf72)* (Brown et al. 2021; Balendra and Isaacs 2018). These mutations drive disease progression through multiple mechanisms, including loss of normal protein function and toxic gain‐of‐function effects such as protein misfolding, RNA foci accumulation and dipeptide repeat protein aggregation.

Antisense oligonucleotides (ASOs) have emerged as a promising therapeutic strategy to directly target these pathogenic transcripts (Abati et al. 2020; McCampbell et al. 2018). By binding complementary RNA sequences, ASOs can modulate gene expression by degrading mRNA, altering splicing, or repressing translation. The recent FDA approval of Tofersen, an ASO that reduces mutant *SOD1* expression in ALS, highlights the clinical potential of this approach (Blair 2023; Everett and Bucelli 2024; Miller et al. 2022). Similarly, ASOs targeting *C9orf72* HRE‐containing RNAs have been advanced in clinical trials, aiming to reduce pathological RNA foci and dipeptide‐repeat protein aggregation while preserving normal transcripts (Crooke 2017, Le Bras 2022; Miller et al. 2013; Smith et al. 2006).

Despite their therapeutic promise, ASO therapies for ALS and other neurological disorders face significant delivery challenges. Achieving effective concentrations in the central nervous system (CNS) typically requires invasive intrathecal or intracerebroventricular administration, which carries risks of infection, discomfort and reduced patient compliance (Hill and Meisler 2021; Miller et al. 2022; Stein and Castanotto 2017). Systemic administration of ASOs, while less invasive, suffers from poor penetration of the restrictive blood–brain barrier (BBB), limited retention in the CNS and off‐target peripheral distribution. In addition, the large size and negative charge of ASOs hinder efficient passive cellular uptake, and their susceptibility to nuclease‐mediated degradation in circulation further compromises their stability and half‐life (Ramasamy et al. 2022; Younis et al. 2022). Collectively, these barriers restrict CNS bioavailability of ASOs and limit their therapeutic efficacy for neurological disorders.

Extracellular vesicles (EVs), nanosized membranous particles released by virtually all cell types, have recently gained attention as natural carriers for nucleic acid therapeutics. As key mediators of intercellular communication, EVs can load and transport bioactive molecules, including proteins, nucleic acids and lipids, between local and distant cells. Their endogenous origin confers high biocompatibility, low immunogenicity and reduced systemic clearance compared to synthetic nanoparticles (Armstrong and Stevens 2018; Johnsen et al. 2018; Kamerkar et al. 2017; Yang et al. 2019). Unlike lipid nanoparticles, which often elicit toxic immune or inflammatory responses and predominantly accumulate in the liver, EVs exhibit favorable biodistribution and safety profiles. Importantly, EV tropism depends on the cell of origin (Fernandez‐Fernandez et al. 2011). For example, EVs derived from hypoxic tumour cells preferentially target tumour tissue (Jung et al. 2018), while CNS‐derived EVs can cross the BBB and selectively interact with neuronal populations (Shi et al. 2019).

Neural stem cell‐derived EVs (NSC‐EVs) are particularly attractive for CNS‐targeted therapies. Previous studies have shown that NSC‐EVs exert neuroprotective and anti‐inflammatory effects in models of traumatic brain injury, Alzheimer's disease, and stroke (Spellicy et al. 2020; Webb et al. 2018). However, whether NSC‐EVs can be harnessed to deliver nucleic acid therapeutics such as ASOs in ALS and other neurological disorders remains an open question. Specifically, it is unclear whether NSC‐EVs can efficiently protect ASOs from degradation, enhance neuronal uptake, and improve CNS targeting via minimally invasive, clinically feasible administration routes.

Here, we demonstrate that human NSC‐EVs can be developed as a platform to efficiently deliver therapeutic ASOs for ALS treatment. We evaluated their neuronal cell uptake in vitro, their biodistribution and stability in vivo following intracerebroventricular, intranasal, or intravenous administration. Finally, we assessed the therapeutic effects in cellular and mouse models of ALS. Our findings establish NSC‐EVs as a versatile and clinically feasible platform to overcome major delivery barriers for ASO therapies, offering a promising strategy to enhance CNS targeting and synergistic therapeutic efficacy of nucleic acid therapeutics in ALS and potentially other neurological disorders.

## 2 Methods and Materials

### 2.1 Cell Culture

Neural stem cells (NSCs) were acquired from Aruna Bio Inc and cultured in Neurobasal media containing 2% neural media supplement, 0.04% bFGF, 0.1% leukaemia inhibitory factor, 1% 2 mM L‐glutamine, and 1% 100 U/mL penicillin–streptomycin. Human fibroblast cells (BJ, CRL‐2522, ATCC) were cultured in MEM containing 10% fetal bovine serum, 100 U/mL penicillin and 100 µg/mL penicillin–streptomycin. The BBB hCMEC/D3 neurovascular cell line was purchased from Millipore Sigma (Massachusetts, US) and cultured in EndoGRO‐MV Complete Culture Media. Human neuroblastoma cells (SH‐SY5Y, CRL‐2266 ATCC) were cultured in AB2 basal neural media containing 2% neural media supplement, 2 mM L‐glutamine, 100 U/mL penicillin–streptomycin and leukaemia inhibitory factor. Fibroblast cells, human mature neurons and neurovascular cells were all cultured under standard conditions at 37°C, 5% CO2 before extracellular vesicle uptake assays.

### 2.2 Animal Use

Adult male wild‐type (C57BL/6J, stock #000664) and ALS transgenic mice (B6SJL‐TgN [SOD1*G93A]1Gur/J, stock #002726) were purchased from the Jackson Laboratory and used in this study. The use of animals was approved by the University of Georgia Institutional Animal Care and Use Committee. The studies complied with the National Institutes of Health Guidelines for the Care and Use of Animals in Research. The animals were maintained in the Department of Animal and Dairy Science's animal facilities at the University of Georgia.

ALS mice were randomly assigned to treatment groups prior to treatment: vehicle (*n* = 4), free ASO (*n* = 5), and NSC‐EV‐ASO (*n* = 6). ALS mice received biweekly intranasal administration of NSC‐EV‐ASOs beginning at postnatal day 60 (2.66 µg ASO, 8.8 × 10^9^ NSC‐EVs per dose), followed by booster doses at days 100 and 105 (6.58 µg ASO, 2.18 × 10^10^ NSC‐EVs per dose). The total dose per mouse was 27.86 µg ASO and 9.22 × 10^10^ NSC‐EVs. Free ASO administered at equivalent doses served as a control. Based on in vivo biodistribution results demonstrating enhanced brain accumulation of NSC‐EV‐ASOs, the ASO dosage was intentionally reduced compared to that used in prior ALS mouse studies (Jiang et al. 2016). This dosing strategy was designed to directly assess the delivery efficiency and therapeutic advantage conferred by EV‐mediated ASO delivery. Behavioural assessments and histological analyses were conducted by investigators blinded to treatment allocation. Animals were excluded only in cases of predefined technical failure, such as unsuccessful intranasal administration or tissue‐processing artifacts. No animals were excluded based on experimental outcomes.

Motor function was assessed weekly beginning at day 60. Grip strength was measured using a digital grip strength meter (Columbus Instruments, DFIS‐2). Rotarod performance was tested using an accelerating rotarod (Ugo Basile) with three trials per session. The neurological deficit score (NDS) was evaluated based on hindlimb weakness, gait abnormalities and tremors, with disease onset defined as an NDS of 1. Body weight was recorded weekly.

### 2.3 ASO Sequences

An ASO targeting human SOD1 was designed based on previously validated sequences (Jin and Zhong 2023). The ASO was synthesized with phosphorothioate backbone modifications and 2′‐O‐methoxyethyl (2′‐MOE) chemistry for enhanced stability. To enable fluorescence tracking, ASOs were conjugated at the 3′ end with Atto‐647N (IBA Lifesciences). A C9orf72 repeat‐targeting ASO was synthesized similarly (Jiang et al. 2016). Lyophilized ASOs were dissolved in sterile nuclease‐free water to a stock concentration of 1 mM and stored at ‐20°C.

### 2.4 EV Isolation and Characterization

Conditioned medium from NSC cultures was collected after 48 h, and EVs were isolated by differential ultracentrifugation, following the guidelines of MISEV 2018 (Théry et al. 2018) and MISEV 2023 (Welsh et al. 2024). Briefly, the media were centrifuged at 300 × g for 10 min, then at 2000 × g for 20 min to remove cells and debris, followed by 10,000 × g for 30 min to eliminate large vesicles. The supernatant was ultracentrifuged at 100,000 × g for 60 min at 4°C using a Beckman Coulter Optima XPN‐100 ultracentrifuge with a SW41Ti rotor. Pelleted EVs were washed once in sterile PBS and centrifuged again at 100,000 × g for 70 min. EVs were resuspended in sterile PBS and quantified by nanoparticle tracking analysis (NTA, NanoSight NS300). EV doses used in all the following uptake and functional experiments were normalized to particle number measured by NTA. For characterization, EV morphology was visualized using transmission electron microscopy (TEM, JEM‐1011). EV protein markers were assessed by Western blotting using antibodies against Alix, Flotillin‐1, Syntenin‐1, CD63 and CD9, while Calnexin served as a negative control. Membrane integrity was evaluated by MemGlow‐488 staining and particle size distribution was measured by nano flow cytometry (NFC, Nano Analyzer). Protein concentration was determined using a bicinchoninic acid (BCA) protein assay kit (Thermo Fisher Scientific).

### 2.5 Liposome Preparation

Liposomes were prepared using the film hydration method following previous publications (Briuglia et al. 2015, Bangham et al. 1965). Briefly, 1,2‐distearoyl‐sn‐glycero‐3‐phosphocholine (DSPC) and cholesterol were dissolved in chloroform at a molar ratio of 7:3. The lipid solution was transferred to a round‐bottom flask, and the organic solvent was removed under reduced pressure using a rotary evaporator to form a thin lipid film on the inner wall of the flask. The lipid film was further dried under vacuum for at least 2 h to remove residual solvent. The dried lipid film was hydrated with sterile PBS and incubated at room temperature with gentle vortexing to generate multilamellar vesicles. The resulting suspension was subsequently extruded through polycarbonate membranes with defined pore sizes (200 nm followed by 100 nm) using a mini‐extruder to produce unilamellar liposomes with a uniform nanoscale size distribution. The size distribution and particle concentration of liposomes were characterized by NTA to confirm physicochemical properties comparable to those of EV preparations. Liposomes were stored at 4°C and used within several days of preparation.

### 2.6 Loading of ASO Into NSC‐EV

Passive loading of ASOs into NSC‐EVs was performed by co‐incubation (Didiot et al. 2016). Purified NSC‐EVs were incubated with fluorescently labelled ASOs at defined ASO‐to‐EV molar ratios (10, 25, 50 and 100k molecules per NSC‐EV) at room temperature for 30–180 min. Unbound ASOs were removed by ultracentrifugation at 100,000 × g for 70 min. Loading efficiency was determined by measuring pellet‐associated fluorescence relative to the initial ASO input using a fluorescence plate reader (Tecan Infinite M200 Pro). For downstream applications, 25k ASO per NSC‐EV and a 90‐min incubation were selected as the optimized loading conditions.

ASO‐associated NSC‐EVs (NSC‐EV‐ASOs) were analyzed by NTA for size distribution and concentration, and Western blotting for EV markers to confirm preservation of structural and molecular integrity after ASO incorporation.

### 2.7 Cellular Uptake Assays

Recipient fibroblasts, mature neurons, and neurovascular endothelial cells were seeded at 60% confluency in a 6‐well plate and cultured for 24 h under standard conditions at 37°C. Standard media were switched to fetal bovine serum‐free media before EV co‐culture. NSC‐EV‐ASOs were administered to cells, which were then co‐cultured for 4 h. After co‐culture, the cells were washed three times with PBS, then dissociated with 0.05% trypsin, and concentrated to approximately 1 million cells per 50 µL for flow cytometry analysis. Using the INSPIRE software, the acquisition was performed on the ImageStreamX Mark II (Luminex Corporation, Seattle, Washington). A minimum of 5000 cell events were acquired. ATTO647N was excited by a 640 nm argon laser at 150 mW, and fluorescence was collected on channel eleven (640‐745 nm). Data analyses were conducted with IDEAS software (Luminex) using the following gating strategy. The bright fields were first gated to eliminate doublets and debris. The gated data were used to create histograms and generate statistical references, measuring the maximum pixel intensity (the intensity of the brightest pixels in an image). Maximum pixel counts were generated using customized IDEAS software templates. Three biological replicates were analyzed, each with three independently acquired technical replicates.

### 2.8 In Vivo Biodistribution Assay

For biodistribution, wild‐type C57BL/6J mice (6–8 weeks old) received a single dose of fluorescently labelled NSC‐EV‐ASOs (*n* = 3), while control animals received an equivalent amount of free ASO prepared in sterile PBS (*n* = 3). The administered dose corresponded to 2.66 µg of ASO (8.8 × 10^9^ EVs) in PBS, consistent with the results of functional validation experiments.

For intracerebroventricular (*i.c.v*.) administration, mice were anesthetized and injected stereotactically into the lateral ventricle with either NSC‐EV‐ASOs or free ASOs (Park et al. 2024). Animals were perfused with PBS on post‐injection days 7 (PD 7) or 21 (PD 21), and major organs (brain, heart, liver, spleen, lungs and kidneys) were harvested for ex vivo fluorescence imaging.

For intranasal (*i.n*.) delivery, mice were lightly anesthetized and administered 2–3 µL drops of NSC‐EV‐ASOs or free ASOs into each nostril until the full 20 µL dose was delivered (Tolomeo et al. 2023). The solution was applied gradually in alternating nostrils to avoid overflow and ensure intranasal retention. At 120 min post‐injection, mice were perfused, and major organs were collected for biodistribution analysis.

For intravenous (*i.v*.) delivery, a single tail‐vein injection of NSC‐EV‐ASOs or free ASOs was performed using a 27G catheter, followed by a saline flush to ensure complete administration (Kang et al. 2023). At 120 min post‐injection, mice were perfused, and major organs were harvested.

Biodistribution was assessed using ex vivo fluorescence imaging with the IVIS Imaging System Lumina II (Newton 7.0, Vilber, France), equipped with a 640 nm excitation filter and a 680 nm emission filter. Fluorescence intensity was quantified as radiant efficiency (photons/s/cm^2^/sr/(µW/cm^2^)) using Living Image software. To evaluate cellular localization within the CNS, brain sections were cryosectioned, immunostained with the neuronal marker NeuN (Millipore, MAB377, 1:500), and counterstained with DAPI, and imaged by confocal microscopy (Zeiss LSM 880). Co‐localization of ASO fluorescence with NeuN‐positive neurons was analyzed to confirm neuronal uptake.

### 2.9 Immunostaining

Brain and spinal cord tissues were collected following transcardial perfusion with PBS and 4% paraformaldehyde, post‐fixed overnight at 4°C, and cryoprotected in 30% sucrose before embedding in OCT compound. Tissue blocks were sectioned at 20–30 µm using a cryostat (Leica CM3050S) and mounted onto Superfrost Plus slides. For immunofluorescence, sections were permeabilized with 0.3% Triton X‐100 in PBS, blocked in 5% normal donkey serum for 1 h at room temperature, and incubated overnight at 4°C with primary antibodies against NeuN (1:500, Millipore) and GFAP (1:500, Dako). After washing, sections were incubated with Alexa Fluor‐conjugated secondary antibodies (1:1000, Thermo Fisher Scientific) for 1 h at room temperature, followed by nuclear counterstaining with DAPI (1 µg/mL). Slides were mounted with antifade medium and imaged on a Zeiss LSM 880 confocal microscope. Quantification of neuronal survival and glial activation was performed by blinded investigators using ImageJ software, with motor neurons counted in the ventral horn of lumbar spinal cord sections and GFAP‐positive areas quantified as a percentage of total tissue area. For colocalization of ASOs, ASO fluorescence was analyzed relative to NeuN‐positive neurons, and Pearson's correlation coefficients were calculated from confocal z‐stacks.

### 2.10 Lysosomal Trafficking and Co‐Localization Analysis

Intracellular trafficking of EV‐delivered ASOs was evaluated using confocal microscopy combined with lysosomal staining (Torres‐Vanegas et al. 2022). Cells were seeded onto glass‐bottom culture dishes and incubated overnight prior to treatment. To visualize lysosomal compartments, cells were stained with LysoTracker Green DND‐26 (Invitrogen, Catalog #L7526) according to the manufacturer's instructions prior to imaging. NSC‐EV‐ASOs were added to cells and incubated at 37°C for the indicated time points.

After staining, cells were washed with PBS and maintained in phenol red‐free imaging medium. Confocal images were acquired using a laser scanning confocal microscope. Co‐localization between NSC‐EV‐ASO fluorescence and lysosomal compartments was quantified using image analysis software by calculating the fraction of ASO fluorescence overlapping with LysoTracker‐positive signals. For each condition, multiple fields of view were analyzed, and the percentage of co‐localized fluorescence was calculated to assess the extent of lysosomal association over time.

### 2.11 Cell Viability Assay (CCK‐8) Assay

Cell viability following different treatments was evaluated using the Cell Counting Kit‐8 (CCK‐8; Dojindo, Catalog #CK04) assay. Cells were seeded in 96‐well plates and allowed to attach overnight. Cells were then treated with NSC‐EV alone, liposome alone, liposome‐associated ASOs (lipo‐ASO), or NSC‐EV‐associated ASOs (NSC‐EV‐ASO). Treatment conditions were normalized to ensure equivalent particle numbers and ASO doses across groups. After incubation for 72 h, CCK‐8 reagent was added to each well according to the manufacturer's protocol, and the plate was incubated at 37°C to allow colour development. Absorbance was measured at 450 nm using a microplate reader. Cell viability was calculated relative to untreated control cells. All experiments were performed with multiple replicates (*n* = 3) for each condition.

### 2.12 RNA Fish

LNA DNA probes were used against the sense RNA hexanucleotide repeat (Exiqon, Inc.). The probe sequence for detecting sense RNA foci is TYE563‐CCCCGGCCCCGGCCCC. All hybridization steps were performed under RNase‐free conditions following previous publications (Todd et al. 2020). Cells were permeabilized in 0.2% Triton X‐100 in PBS for 10 min at room temperature. The sections were heated to 66°C for 1 h in a hybridization buffer. The cells were then hybridized with denatured probes at 66°C overnight. Slides were washed at room temperature in 0.1% Tween‐20/2×SCC for 5 min and then three times in stringency washes in 0.1× SCC at 65°C for 10 min each.

### 2.13 Quantitative PCR

Total RNA was extracted from cultured cells using the RNeasy Mini Kit (Qiagen) according to the manufacturer's protocols. RNA concentrations were assessed using a NanoDrop spectrophotometer. Reverse transcription was performed with the GoScript Reverse Transcription System (Promega) using 500 ng of total RNA per reaction. Real‐time qPCR was conducted on a QuantStudio 5 Real‐Time PCR System (Applied Biosystems) using Precision PowerTrack SYBR Green Master Mix (PrimerDesign) in a reaction volume of 10 µL, containing 10 nM of each primer and 1 µL of cDNA. Cycling parameters were as follows: initial denaturation at 95°C for 2 min, followed by 40 cycles of 95°C for 15 seconds and 60°C for 1 min. Relative expression levels were calculated using the 2^(‐ΔΔCt) method, normalized to the geometric mean of two reference genes. For analysis of repeat‐containing transcripts in the C9‐HRE model, we adopted TaqMan primer/probe mixes for C9‐HRE, while C9orf72 expression was measured using an independent primer pair spanning exon 2 (Jiang et al. 2016). All quantitative analyses were performed in triplicate biological replicates, with each replicate including technical triplicates.

### 2.14 Western Blot

EVs, cells, or tissues were lysed in RIPA buffer supplemented with protease and phosphatase inhibitors (Roche), and protein concentrations were determined by BCA assay (Thermo Fisher Scientific). Equal amounts of protein (20–40 µg) were resolved by SDS‐PAGE on 4%–12% Bis‐Tris gels (Invitrogen) and transferred to PVDF membranes (Millipore) using a wet transfer system. Membranes were blocked with 5% non‐fat milk in Tris‐buffered saline containing 0.1% Tween‐20 (TBST) for 1 h and incubated overnight at 4°C with primary antibodies, including anti‐SOD1 (1:1000, Abcam), anti‐GFP (1:2000, Santa Cruz), anti‐CD63 (1:1000, System Biosciences), anti‐Alix (1:1000, Cell Signalling Technology), anti‐Syntenin‐1 (1:1000, Abcam), and anti‐GAPDH (1:5000, Abcam). After washing with TBST, membranes were incubated with HRP‐conjugated secondary antibodies (1:5000, Jackson ImmunoResearch) for 1 h at room temperature. Protein bands were visualized using enhanced chemiluminescence (ECL, Pierce) and imaged with a ChemiDoc MP system (Bio‐Rad). Densitometric analysis was performed using Image Lab software. For EV characterization, equivalent numbers of particles, as quantified by nanoparticle tracking analysis, were loaded per lane to allow direct comparison across conditions.

### 2.15 Nuclease Protection Assay

To evaluate the protection of ASO from nuclease degradation, samples were treated with DNase I (1 µM, Roche, Catalog #10104159001) and then incubated at 37°C for 1 h as previously described (Ou et al. 2026). EDTA (500 mM) was added to stop DNase I activity after incubation. SDS (to a final concentration of 1%) was then used to release DNA from the complexes before analysis using SplintR‐qPCR Assay as described below. The integrity of the ASO in each formulation was compared with that of the untreated ASO as a control. NSC‐EV‐ASO preparations used for both in vitro and in vivo functional studies were not subjected to nuclease pre‐treatment prior to administration.

### 2.16 SplintR‐qPCR Assay

Intact ASOs were quantified using a SplintR ligation‐based qPCR assay as previous publication (Jepp et al. 2026; Shin et al. 2022) with minor modifications. Briefly, probes A and B were designed to hybridize adjacent to each other on the target ASO sequence. Probe B contained a 5′ phosphate group required for enzymatic ligation. All ligation probes, primers and double‐quenched probes were synthesized by Integrated DNA Technologies.

For the ligation reaction, the hybridization mixture (12 µL total) contained 2 µL of sample or ASO standard (10 µM to 10 fM), 2 µL of 10× SplintR ligase reaction buffer (New England Biolabs), 2 µL probe A (100 nM), 2 µL probe B (100 nM) and nuclease‐free water. For biological matrices, 2 µL of serum or tissue lysate was included to match the sample conditions. The mixture was heated to 95°C for 5 min, then cooled to 37°C at 0.1°C/s to allow probe hybridization to the target ASO. Subsequently, 8 µL of diluted SplintR ligase (2.5 U per reaction; up to 25 U for fully 2′‐O‐MOE‐modified ASOs) was added, and ligation was carried out at 37°C for 30 min. The ligase was heat‐inactivated at 65°C for 20 min.

Real‐time qPCR was conducted on a QuantStudio 5 Real‐Time PCR System (Applied Biosystems) using Precision PowerTrack SYBR Green Master Mix (PrimerDesign) under the following cycling conditions: initial denaturation at 95°C for 3 min, followed by 40 cycles of 95°C for 10 s and 60°C for 30 s.

For analysis of brain samples, tissues were homogenized in lysis buffer (10 mM Tris‐HCl pH 7.4, 150 mM NaCl, 1% NP‐40, 0.5% sodium deoxycholate, 0.1% SDS, 5 mM EDTA and 1 mM EGTA) (Jepp et al. 2026) and incubated on ice for 60 min with occasional vortexing. The lysates were centrifuged at 16,000 × g for 30 min at 4°C, and the supernatant was collected and transferred to a new microcentrifuge tube. The clarified lysate was aliquoted into 10 µL volumes and stored at −80°C until use. Vehicle‐treated samples were included as baseline controls, and relative intact ASO‐associated signal was calculated using the vehicle group as the baseline reference and the same normalization approach across all experimental groups.

Probe A: 5′ CTCGACCTCTCTATGGGCAGTCACGACAGAGCTGTAGAA 3′
Probe B: 5′ pATGTATCCTGCGCTGAGTCGGAGACACGCAGGGCTTAA 3′
Forward Primer: 5′ GCTCGACCTCTCTATGGGC 3′
Reverse Primer: 5′ TTAAGCCCTGCGTGTCTCC 3′

### 2.17 Statistics Analysis

Statistical analyses were performed on all quantitative data using GraphPad Prism 9.1.1 (San Diego, California). All data were analyzed in triplicate and presented as mean ± standard error of the mean (SEM). Statistical significance was determined using a one‐way or two‐way analysis of variance (ANOVA) with Tukey's or Dunnett's multiple comparison post hoc test compared with controls when appropriate. Disease onset curves were analyzed using the log‐rank (Mantel–Cox) test. *p* < 0.05 was considered significant.

## 3 Results

### 3.1 Loading of Therapeutic ASOs Into NSC‐EVs

**FIGURE 1:**
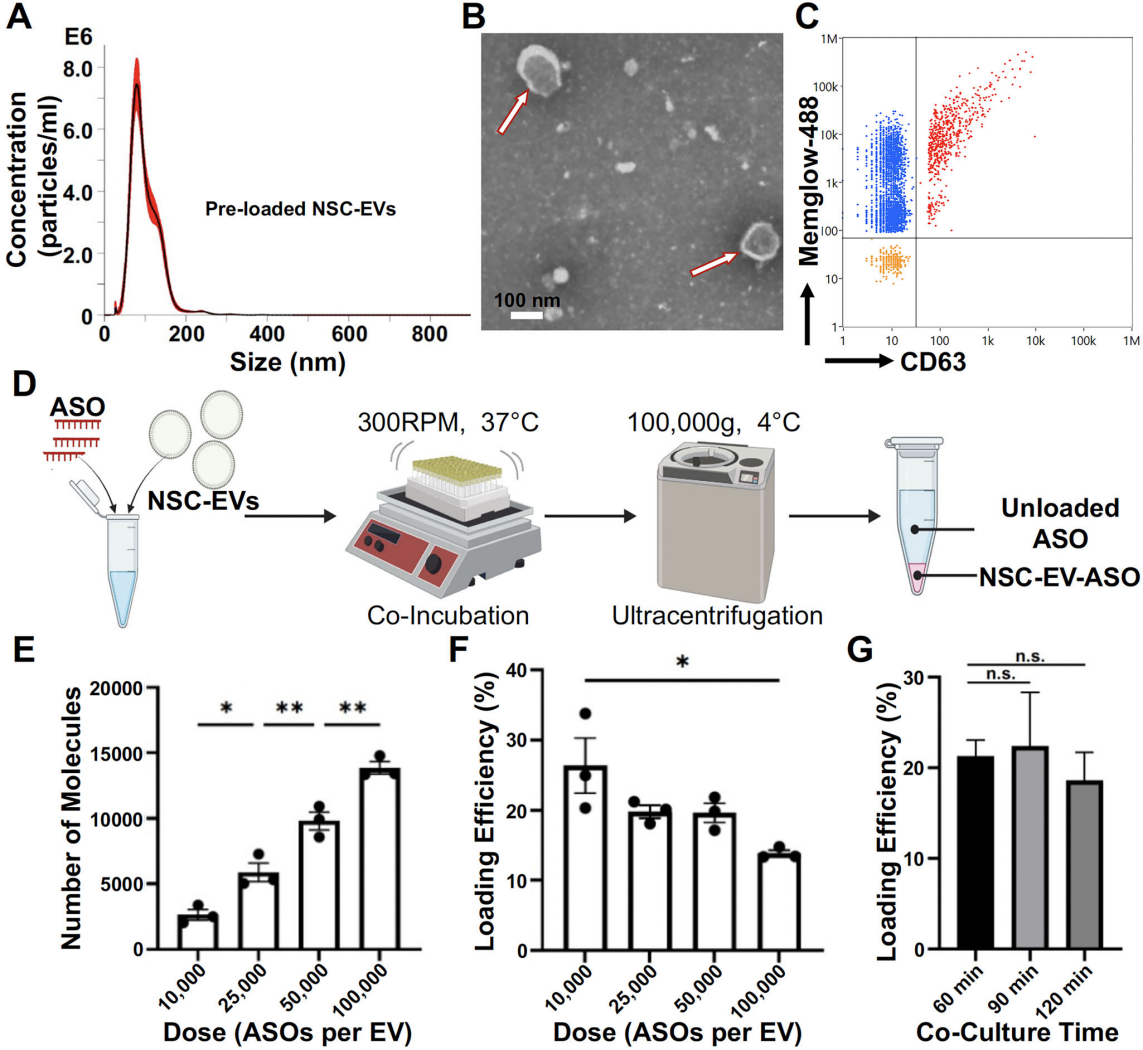
NSC‐EV isolation, characterization, and loading with therapeutic ASOs. (A) Nanoparticle tracking analysis (NTA) of freshly isolated NSC‐EVs shows a predominant particle size distribution centred around 100 nm. (B) Representative transmission electron microscopy (TEM) image depicting the characteristic bilayer membrane morphology of NSC‐EVs. (C) EV integrity and marker expression at the single‐particle level were assessed by nano‐flow cytometry (NFC) using MemGlow‐488 membrane staining and PE‐conjugated anti‐CD63 antibody labelling. (D) Schematic illustration of the passive ASO loading strategy. NSC‐EVs were co‐incubated with fluorescently labelled ASOs for 90 min at 37°C, followed by ultracentrifugation to remove unbound ASOs. The resulting ASO‐associated NSC‐EVs (NSC‐EV‐ASO) were resuspended in PBS for downstream experiments. (E) Dose‐response analysis of ASO loading into NSC‐EVs at different ASO‐to‐EV molar ratios (10, 25, 50 and 100k molecules per EV). The fluorescence intensity associated with EVs was quantified to estimate the ASO content per vesicle. (F) Loading efficiency is calculated as the percentage of EV‐associated ASO fluorescence relative to the initial ASO input. (G) Effect of co‐incubation duration on loading efficiency, showing minimal time‐dependent variation. Each dot represents a biological replicate, which consists of three technical replicates. Statistical significance was determined using one‐way ANOVA with Tukey's post hoc test. * *p* <0.05, ** *p* <0.01, *** *p* <0.001. n.s., not significant.

Given the brain‐targeting and therapeutic potential of neural stem cell‐derived extracellular vesicles (NSC‐EVs) (Madhu et al. 2024; Tian et al. 2021), we explored their potential as a delivery shuttle for ASO‐mediated therapy to enhance its targeting and efficacy for ALS. We first isolated EVs from neural stem cell (NSC) (Hoffrogge et al. 2006; Song et al. 2019) conditioned media using differential ultracentrifugation (Liu et al. 2022). Nanoparticle tracking analysis (NTA) showed a predominant population of particles with a diameter of approximately 130 nm (Figure 1A, Table 1). Transmission electron microscopy confirmed their characteristic bilayer lipid membrane morphology (arrows, Figure 1B). MemGlow‐488 membrane staining demonstrated that approximately 92% of particles retained an intact lipid bilayer (Figure 1C), and all CD63^+^ particles were positive for MemGlow‐488, indicating that the isolation method preserved EV structural integrity. Western blotting revealed enrichment of canonical EV markers, including ALG‐2‐interacting protein X (Alix), Flotillin‐1, Syndecan‐binding protein (Syntenin‐1), and Cluster of Differentiation 9 (CD9), and the absence of the intracellular protein Calnexin (endoplasmic reticulum chaperone protein), confirming high‐purity with a consistent particle‐to‐total protein ratio of 7.83E + 11 (Figure S1A).

**TABLE 1:** NSC‐EV characterization.

Parameters	NSC‐EV
Mode particle size by NTA (nm)	135.1
d10 particle size by NTA (nm)	114.6
d50 particle size by NTA (nm)	146.3
d90 particle size by NTA (nm)	196.5
Particle concentration by NTA (particle/mL)	6.40E+11
On CD81 captured particles, %CD63 positive	85%
Total protein concentration (mg/mL)	0.8169
Particle‐to‐total protein ratio (particle/mg)	7.83E+11

We then synthesized an ASO targeting the ALS‐associated mutant SOD1 and conjugated it with fluorescent Atto‐647 at the 3′ end for visualization (Figure S2) (Jin and Zhong 2023). This ASO has been previously validated for robust suppression of mutant SOD1 protein in the SOD1‐G93A ALS mouse model and clinical trials by ELISA assay (McCampbell et al. 2018, Miller et al. 2022). To load ASO into NSC‐EVs, we employed a non‐invasive, passive co‐incubation method in which fluorescently labelled ASOs were mixed with purified EVs while shaking (Figure 1D). Following ultracentrifugation to separate free ASOs from EV‐associated ones, loading efficiency was calculated by comparing fluorescence in the EV pellet to the initial ASO input. In a dose‐response assay, ASO: EV molar ratios of 10, 25, 50 and 100 k molecules per EV were evaluated. Although the absolute ASO content per EV increased with higher ratios (Figure 1E), loading efficiency peaked at ∼25% at a 10 k ASO/EV ratio and declined at higher ratios (Figure 1F). Variation in co‐incubation time had minimal impact on loading efficiency (Figure 1G). Based on these results, we selected a ratio of 25 k ASO per EV and a 90‐min incubation as the optimal condition, balancing loading yield with material efficiency for downstream applications.

Importantly, NTA confirmed that ASO‐associated NSC‐EVs (NSC‐EV‐ASO) retained a unimodal size distribution with a peak diameter of approximately 95 nm (Figure S1B), comparable to unloaded EVs (Figure S1C). Western blotting further showed no alteration in canonical EV marker expression after ASO loading (Figure S1A). These observations support the notion that the non‐invasive passive loading strategy is an efficient method for packaging ASOs into EVs, which does not compromise the size, structural integrity, and molecular characteristics of the EVs.

To further evaluate the stability and integrity of EV‐associated ASOs, we performed nuclease protection experiments followed by SplintR‐qPCR analysis (Figure S3). Specifically, NSC‐EV‐ASO preparations were treated with DNase, and the digested mixture was quantified using SplintR‐qPCR, which specifically detects full‐length ASO molecules. We observed that a substantial fraction (74.2% ± 1.8%) of the EV‐associated ASO signal remained detectable after DNase treatment, relative to the corresponding EV‐associated ASO signal without DNase treatment, whereas free ASOs completely degraded and became undetectable. In contrast, when NSC‐EV‐ASO samples were treated with detergent prior to DNase digestion, the ASO signal was abolished. These results indicate that a significant portion of the EV‐associated ASO was protected from nuclease degradation under the tested conditions, consistent with protection by intact EV membranes.

### 3.2 NSC‐EVs Enhanced Cellular Uptake of Therapeutic ASOs

**FIGURE 2:**
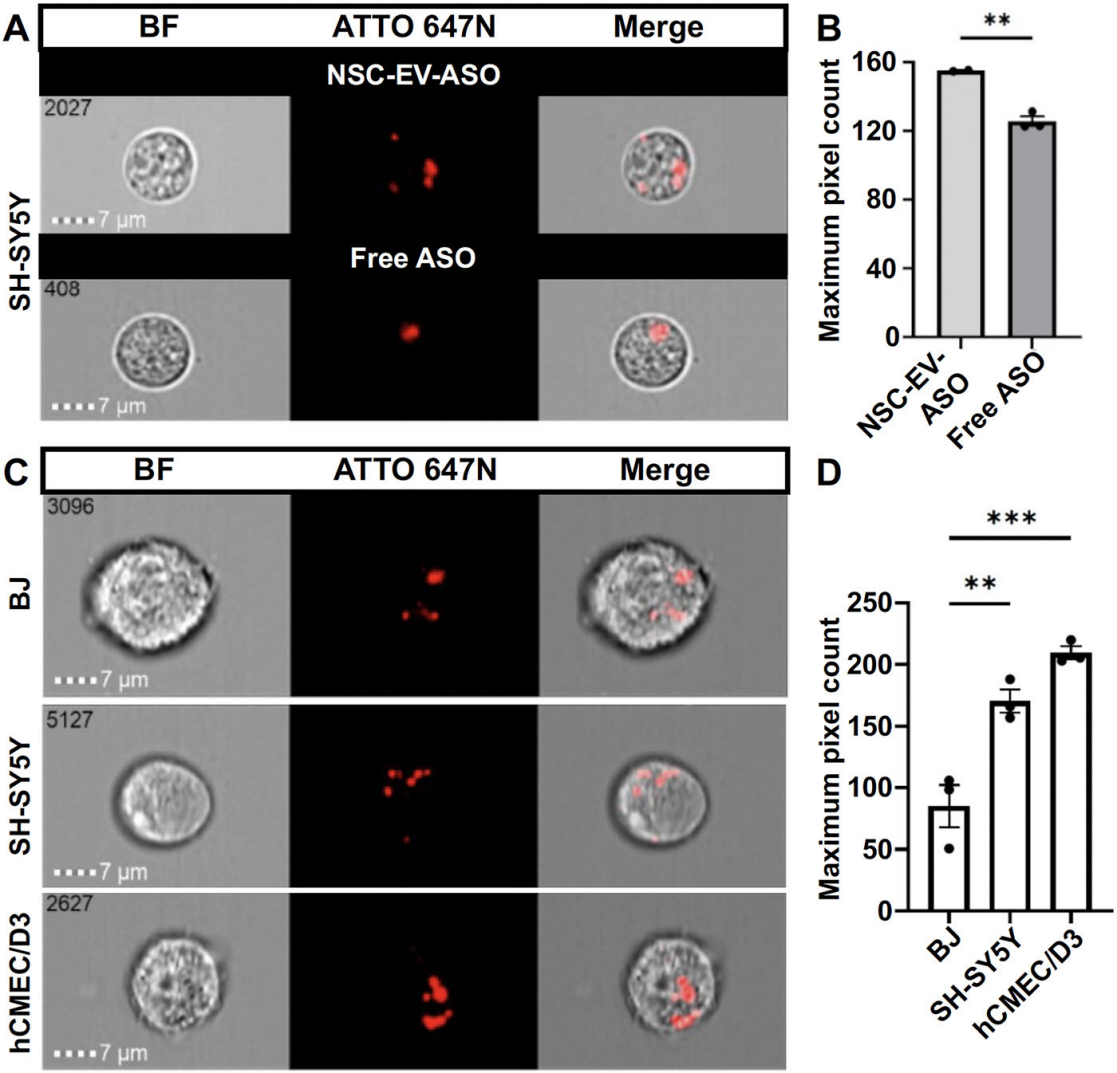
NSC‐EVs enhanced cellular uptake of therapeutic ASOs. (A) Representative imaging flow cytometry images of SH‐SY5Y cells incubated with either free fluorescently labelled ASOs (Atto‐647N) or NSC‐EV‐associated ASOs (NSC‐EV‐ASO) for 4 h. (B) Quantification of intracellular ASO fluorescence in SH‐SY5Y cells, represented by maximum pixel intensity per cell, reveals significantly increased uptake in the NSC‐EV‐ASO group compared to free ASOs. (C) Representative imaging flow cytometry plots of ASO uptake across three cell types: neurons (SH‐SY5Y), neurovascular cells (hCMEC/D3, an in vitro blood‐brain barrier model), and non‐neuronal fibroblasts (BJ). (D) Quantitative analysis of maximum pixel intensity demonstrates preferential uptake of NSC‐EV‐ASOs by neurons and neurovascular cells compared to fibroblasts. Each dot represents a biological replicate, which consists of three technical replicates. Statistical significance was determined using one‐way ANOVA with Tukey's post hoc test. * *p* <0.05, ** *p* <0.01, *** *p* <0.001. n.s., not significant.

To determine whether NSC‐EVs enhance the cellular uptake of therapeutic ASOs, we first performed uptake assays in disease‐relevant neuronal cells. Human neuroblastoma cells (SH‐SY5Y) were incubated with either NSC‐EV‐ASOs or an equal amount of free ASOs for four hours. After incubation, the culture medium was removed, and the cells were subjected to multiple PBS washes, followed by trypsinization. The use of trypsin not only detached the cells but also reduced potential interference from surface‐bound or nonspecifically associated ASOs, thereby allowing subsequent measurements to reflect intracellular uptake predominantly. Imaging flow cytometry was then applied to visualize and quantify the fluorescently labelled ASOs in trypsin‐treated recipient cells, as previously reported (Jurgielewicz et al. 2020). Compared with free ASOs, NSC‐EV‐mediated delivery significantly increased the uptake of fluorescent (ATTO‐647N)‐tagged ASOs (Figure 2A), as evidenced by higher intracellular fluorescence indicated by a marked increase in the maximum pixel intensity per cell (Figure 2B).

We then assess the cell‐type specificity of NSC‐EV‐mediated ASO delivery by comparing cellular uptake of ASOs across three different cell types: neurons (SH‐SY5Y), neurovascular cells (hCMEC/D3, an in vitro model of the BBB), and non‐neuronal control (BJ human foreskin fibroblast cell line from ATCC). Consistent with the reported tropism of NSC‐derived EVs (Ellison et al. 2021), we found that ASO uptake was significantly higher in neuronal and neurovascular cells compared to fibroblasts (Figure 2C). Quantitative analysis revealed approximately 1.5‐fold and 2‐fold higher maximum pixel intensities in neurons and neurovascular cells, respectively (Figure 2D). Together, these results demonstrate that NSC‐EVs not only enhance cellular uptake of ASOs but also preferentially deliver cargos to CNS‐relevant cell types, supporting their potential as a precision therapeutic platform for ALS.

To further examine the intracellular trafficking of EV‐delivered ASOs, we visualize lysosomal compartments using LysoTracker staining and evaluate the spatial relationship between NSC‐EV‐ASO signals and lysosomes (Figure S4). At 70 min post‐treatment, a substantial portion of NSC‐EV‐ASO fluorescence (73.2% ± 13.2%) co‐localized with LysoTracker‐positive compartments, indicating that the majority of EV‐delivered ASOs are trafficked to the endo‐lysosomal pathway following cellular uptake. Over time, NSC‐EV‐ASO signals increasingly appeared to dissociate from lysosomal compartments, with the co‐localization percentage decreasing to 38.4% ± 10.5% at 90 min post‐treatment. These observations suggest that a fraction of EV‐delivered ASOs may escape endo‐lysosomal confinement over time.

### 3.3 NSC‐EV‐mediated delivery improved CNS‐targeting of ASOs in vivo

**FIGURE 3:**
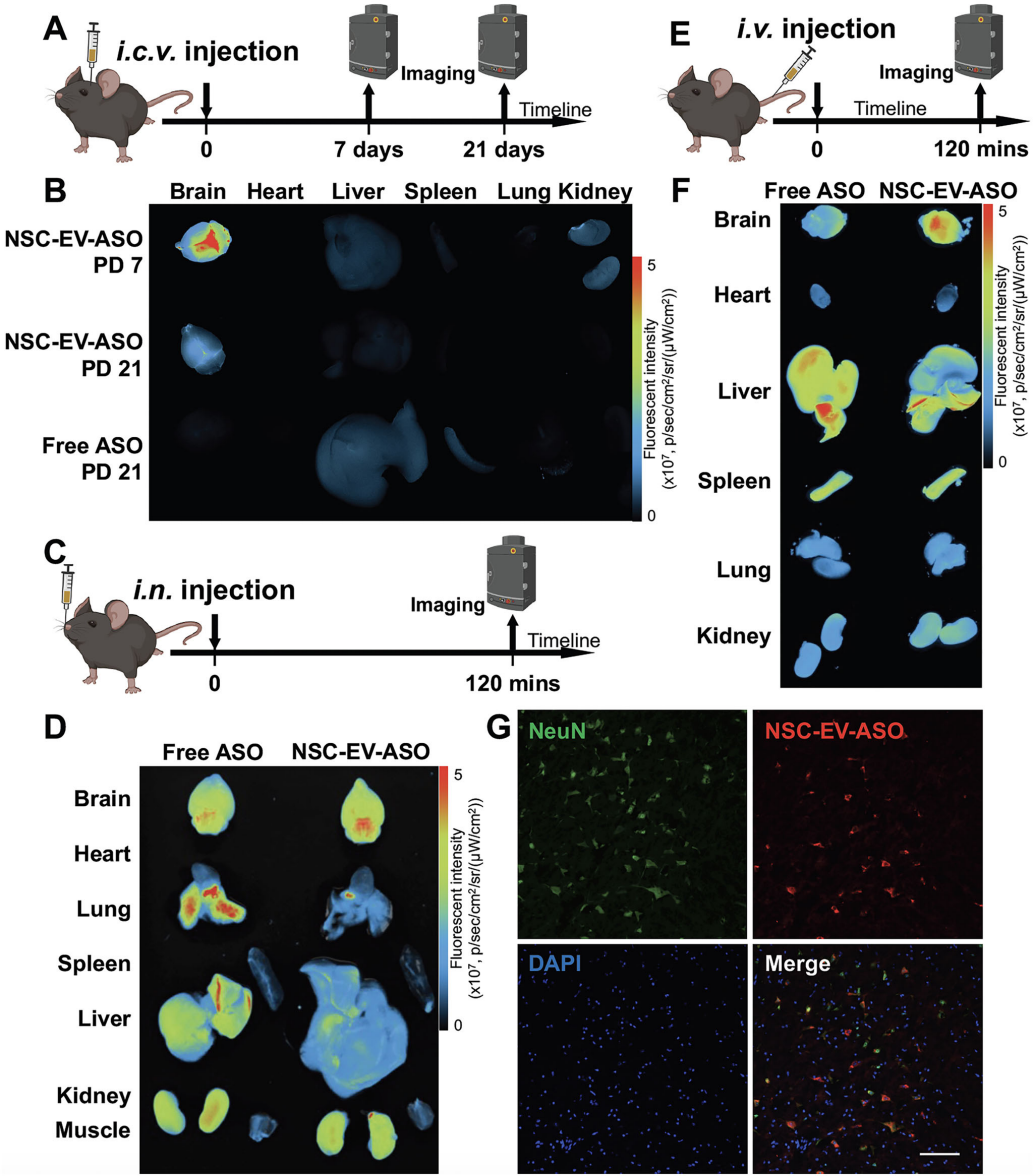
NSC‐EV‐mediated delivery improved CNS‐targeting of ASOs in vivo. (A) Schematic of intracerebroventricular (i.c.v.) administration of NSC‐EV‐associated ASOs (NSC‐EV‐ASO) or free ASOs in wild‐type mice, followed by tissue collection at post‐injection day (PD) 7 and PD21. (B) Representative images of major organs showing the biodistribution of fluorescently labelled ASOs at PD7 and PD21. NSC‐EV‐ASOs exhibited prolonged retention in the brain with minimal accumulation in peripheral organs compared to free ASOs. (C) Schematic of intranasal (i.n.) administration routes for NSC‐EV‐ASOs versus free ASOs. (D) Representative images of organ biodistribution following i.n. administration at 120 min. NSC‐EV‐ASOs showed robust brain accumulation with limited peripheral distribution, whereas free ASOs exhibited negligible CNS uptake. (E) Schematic of intravenous (i.v.) administration routes for NSC‐EV‐ASOs versus free ASOs. (F) Representative images and quantification of ASO biodistribution following i.v. administration, demonstrating enhanced brain accumulation in the NSC‐EV‐ASO group compared to free ASOs, while maintaining systemic distribution. (Gs) Immunofluorescence staining of brain sections showing co‐localization of fluorescent ASO signals with the neuronal marker NeuN (green) and nuclei (DAPI, blue), confirming neuronal targeting by NSC‐EV‐ASOs in vivo. Scale bar in G, 200 µm. *N* = 3 in each group were analyzed.

We next evaluated the ability of NSC‐EVs to improve the CNS‐targeting efficiency of ASOs in vivo by comparing the biodistribution of EV‐associated ASOs with free ASOs administered through different routes. First, we performed intracerebroventricular (*i.c.v*.) injection in wild‐type mice. Animals received either a single dose of NSC‐EV‐ASOs or an equivalent amount of free ASOs as controls. At 7 and 21 days post‐injection, mice were perfused, and major organs were collected to assess the biodistribution of fluorescently labelled ASOs (Figure 3A). On post‐injection day 7 (PD 7), ASO‐associated fluorescence signals were predominantly retained in the brain, with minimal signals observed in peripheral organs such as livers and kidneys (Figure 3B). Notably, NSC‐EV‐ASO‐associated fluorescence signals persisted in the brain up to 21 days (PD 21), whereas signals from free ASOs were largely undetectable. These results indicate that NSC‐EVs shield ASOs from rapid clearance and extend their retention in the CNS, potentially prolonging therapeutic exposure.

Considering that minimally invasive delivery routes are more favourable for clinical translation due to lower safety risks and improved patient compliance, we further assessed the biodistribution of ASOs following intranasal (*i.n*.) and intravenous (*i.v*.) administrations. Following a single *i.n*. dose (Figure 3C), NSC‐EV‐ASOs exhibited strong brain accumulation, whereas free ASOs showed little CNS uptake at 120 min post‐injection, as indicated by fluorescence signals (Figure 3D). In contrast, *i.v*. administration of NSC‐EV‐ASOs led to significantly stronger fluorescence signals in the brain compared to free ASOs, while also maintaining a systemic distribution profile (Figure 3E–F). These data suggest that while both *i.n*. and *i.v*. routes leverage the CNS tropism of NSC‐EVs, but with distinct delivery profiles: *i.v*. achieves broader CNS distribution, whereas *i.n*. favours more regionally focused targeting.

To further examine the contribution of EV origin to CNS targeting, EVs derived from HEK 293T cells (293T‐EVs) were used as a non‐neural EV control. Both NSC‐EVs and 293T‐EVs were incubated with fluorescently labelled ASOs using the same loading condition and administered via *i.v*. injections. At 120 min after administration, animals exhibited higher brain‐associated fluorescence in mice treated with NSC‐EV‐ASOs than in those treated with 293T‐EV‐ASOs (Figure S5). These findings suggest that EV cellular origin may contribute to differences in brain biodistribution under the conditions examined.

Finally, to examine the cellular localization of therapeutic ASOs delivered by NSC‐EVs, brain sections were stained for the neuronal marker NeuN and nuclear stain DAPI. Fluorescent ASO signals co‐localized predominantly with NeuN‐positive neurons (Figure 3G), confirming that NSC‐EVs efficiently deliver ASOs to neuronal populations in vivo. These data demonstrate that NSC‐EVs enhance brain delivery across multiple routes, highlighting *i.n*. administration as a non‐invasive and clinically promising strategy for EV‐based ASO therapies.

### 3.4 NSC‐EV‐ASOs Suppressed Pathogenic Genetic Factors in ALS Cellular Models

**FIGURE 4:**
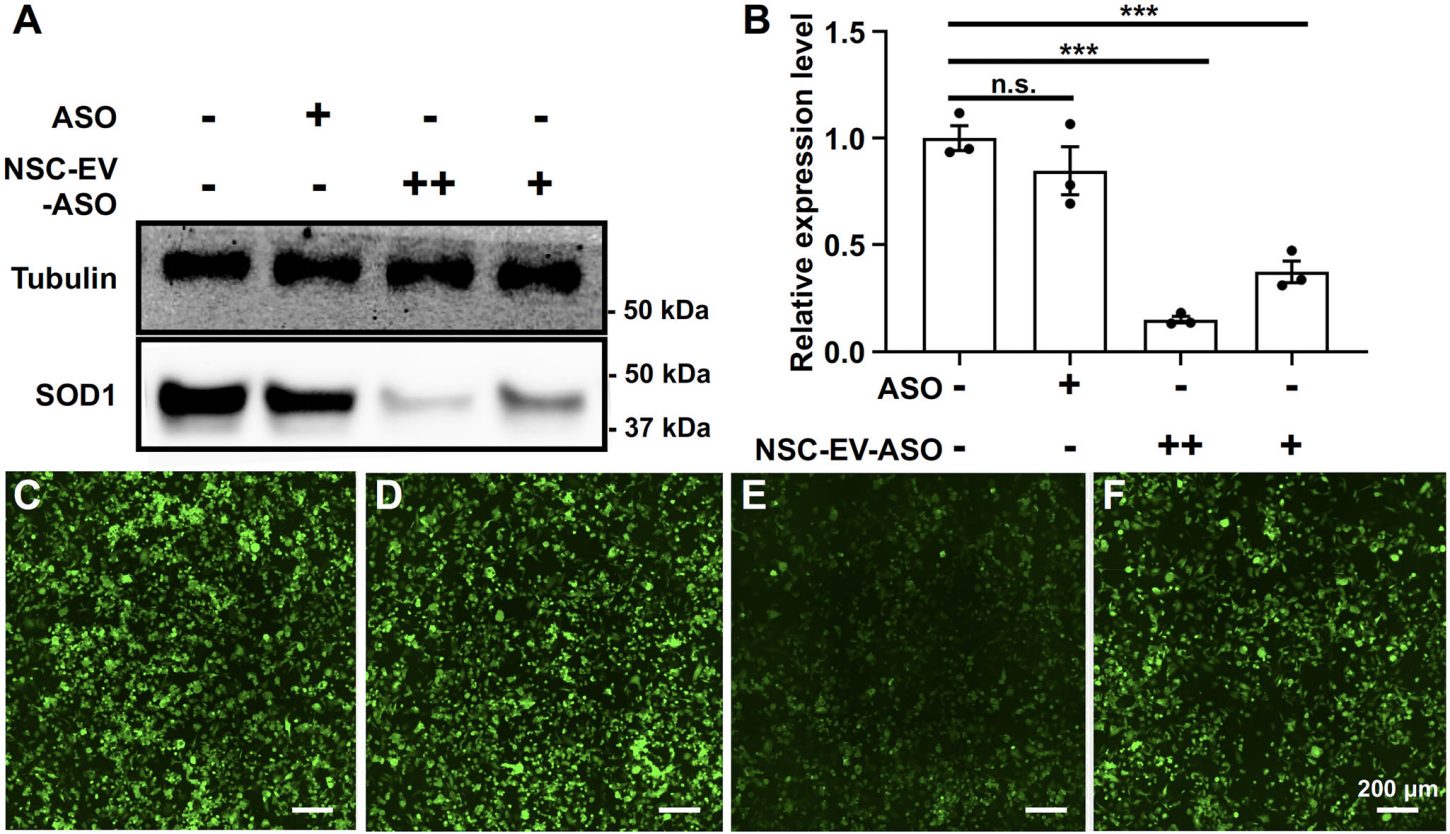
NSC‐EV‐ASOs suppressed pathogenic genetic factors in ALS cellular models. (A) Western blot analysis of SOD1 protein levels in NSC‐34 cells overexpressing mutant SOD1‐GFP after treatment with vehicle, free ASOs, or NSC‐EV‐ASOs (low or high dose) for 48 h. (B) Quantification of Western blot assay. (C‐F) Representative fluorescence images showing SOD1‐GFP aggregates in NSC‐34 cells treated with (C) vehicle, (D) free ASO, (E) high‐dose NSC‐EV‐ASO, (F) low‐dose NSC‐EV‐ASO. Each dot represents a biological replicate, which consists of three technical replicates. Statistical significance was determined using one‐way ANOVA with Tukey's post hoc test. * *p* <0.05, ** *p* <0.01, *** *p* <0.001. n.s., not significant.

ASOs regulate gene expression by promoting target RNA degradation, altering splicing, or interfering with protein synthesis (Ramasamy et al. 2022). To evaluate the functional efficacy of NSC‐EV‐ASO complexes, we first tested NSC‐EV‐ASOs targeting human *SOD1* in the NSC‐34 cell line (a mouse motor neuron‐like hybrid cell line), engineered to overexpress doxycycline‐inducible GFP‐tagged mutant SOD1 (G93A), a well‐established cellular ALS model (Zhou et al. 2023) (Figure S6A). Following 48 h of treatment with either NSC‐EV‐ASO or free ASOs, fluorescent imaging confirmed efficient cellular uptake and retention of ASOs in NSC‐34 cells (Figure S6B). The ASO‐associated fluorescent signals displayed a punctate intracellular distribution that was largely non‐overlapping with the diffuse cytosolic SOD1‐GFP signal (Figure S6C,D). Notably, cells displaying robust ASO uptake showed a weak or non‐detectable SOD1‐GFP fluorescence signal, indicating effective suppression of the target protein. Consistently, western blot analysis revealed a more pronounced reduction in SOD1 protein levels in cells treated with NSC‐EV‐ASOs than in cells treated with free ASO at equivalent doses (Figure 4A,B). Fluorescence imaging further validated these results, showing a marked reduction in SOD1‐GFP fluorescence intensity in cells treated with NSC‐EV‐ASOs (Figure 4E) relative to vehicle control (Figure 4C) or free ASOs (Figure 4D). The degree of knockdown correlated with ASO accumulation at the single‐cell level (Figure S6D) and was dose‐dependent (Figure 4E,F), supporting the enhanced therapeutic potency of NSC‐EV‐mediated ASO delivery.

Considering NSC‐EVs themselves possess intrinsic biological activities (Narayanappa et al. 2025), including reported neuroprotective and anti‐inflammatory effects, we next evaluated whether these intrinsic effects contributed to the observed cellular responses. To control for this possibility, liposomes were prepared to approximate key physicochemical characteristics of EVs, including comparable nanoscale particle size and ASO loading strategy, enabling a controlled comparison between delivery platforms. Specifically, ASOs were loaded into liposomes using a procedure analogous to that for EV loading to generate liposome‐ASO complexes (lipo‐ASO). Cell viability of ALS‐like NSC‐34 cells overexpressing SOD1^G93A^ was measured following treatments with NSC‐EV‐only, liposome‐only, lipo‐ASO, and NSC‐EV‐ASOs under matched dosing conditions normalized for particle number and ASO concentration. NSC‐EV treatment alone produced a modest but measurable effect relative to liposome‐only controls (Figure S7), consistent with previously reported intrinsic bioactivity of NSC‐EVs (Narayanappa et al. 2025). Importantly, NSC‐EV‐ASO treatment produced a significantly stronger effect than either EV‐only or lipo‐ASO treatment, suggesting that the enhanced activity arises from a cooperative interaction between the EV carrier and the ASO cargo.

We next extended this approach to target the aberrant *C9orf72* hexanucleotide repeat expansion (C9‐HRE), the most common genetic cause of familial ALS. ASOs targeting C9‐HRE transcripts (ASO‐C9) were synthesized, loaded into NSC‐EVs (Jiang et al. 2016), and tested in fibroblasts derived from ALS patients carrying the C9‐HRE mutation. Quantitative Reverse Transcription PCR (RT‐qPCR) revealed that NSC‐EV‐ASO‐C9 treatment resulted in a more pronounced reduction in C9‐HRE transcripts compared to an equivalent dose of free ASOs (Figure S8A). Notably, C9orf72 expression measured with an independent primer pair spanning exon 2 was unaffected (Figure S8B), confirming selective depletion of repeat‐containing transcripts rather than global reduction of *C9orf72*. Consistently, RNA fluorescence in situ hybridization (FISH) confirmed that NSC‐EV‐ASO‐C9 significantly reduced the number of pathogenic nuclear RNA foci triggered by aberrant C9‐HRE RNAs in patient fibroblasts, whereas free ASOs at equivalent doses were notably less effective (Figure S8C,D). Collectively, these results demonstrate that NSC‐EV‐mediated delivery enhances intracellular ASO bioavailability and target engagement, enabling efficient suppression of disease‐driving SOD1 and C9‐HRE transcripts in ALS cellular models.

### 3.5 NSC‐EV‐ASOs Delayed Disease Onset and Alleviated Motor Defects in the ALS Mouse Model

**FIGURE 5:**
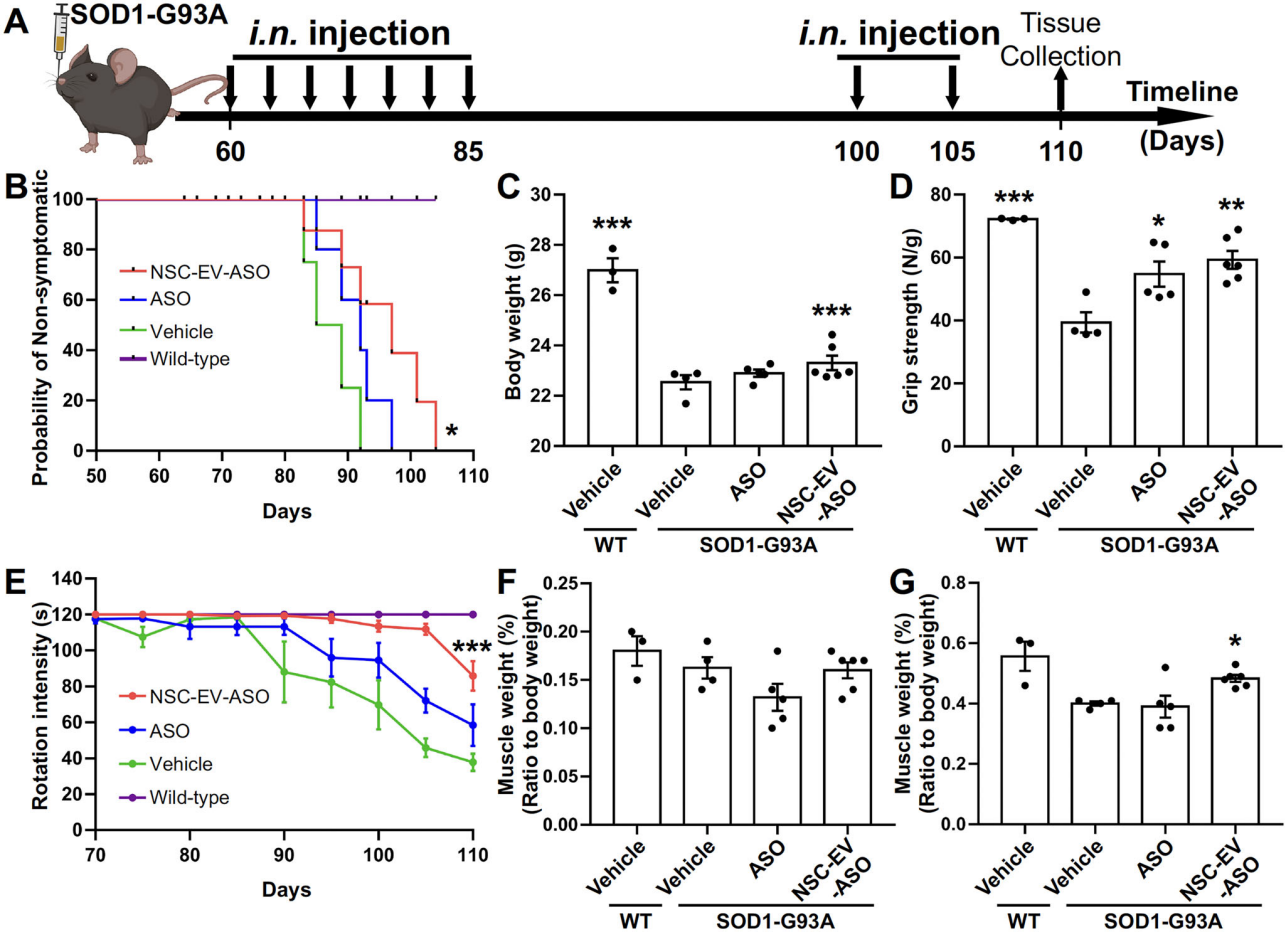
NSC‐EV‐ASOs delayed disease onset and alleviated motor defects in the ALS mouse model. (A) Experimental timeline of intranasal NSC‐EV‐ASO or free ASO administration in SOD1‐G93A mice. Treatments were given biweekly from P60 to P85, followed by booster doses at P100 and P105. (B) Kaplan‐Meier curves showing delayed disease onset in NSC‐EV‐ASO‐treated mice compared with vehicle and free ASO groups. (C) Body weight analysis at P110, showing no significant group differences. (D) Grip‐strength measurements demonstrated significantly improved performance in NSC‐EV‐ASO‐treated mice compared with vehicle‐ and free‐ASO‐treated groups. (E) Rotarod test results indicate preserved motor coordination in the NSC‐EV‐ASO group. (F‐G) Muscle mass analysis showing no significant difference in tibialis anterior (TA, F) weight but significant preservation of gastrocnemius (GAS, G) weight in NSC‐EV‐ASO‐treated mice. Data are presented as mean ± SEM. Wild‐type (*n* = 3), vehicle‐treated (*n* = 4), ASO‐treated (*n* = 5), and NSC‐EV‐ASO‐treated (*n* = 6) mice were analyzed. Statistical significance was determined using the log‐rank (Mantel‐Cox) test for panel B, two‐way ANOVA with Tukey's multiple comparison test for panel E, and one‐way ANOVA with Tukey's post hoc test for panels C, D, F and G. * *p* <0.05, ** *p* <0.01, *** *p* <0.001.

To assess the therapeutic potential of NSC‐EV‐ASO complexes in vivo, we used the SOD1‐G93A ALS mouse model. SOD1‐G93A mice received intranasal administration of either NSC‐EV‐ASOs or an equivalent amount of free ASOs, totalling ∼ 28 µg/mouse, delivered in two series (Figure 5A): biweekly injections from postnatal day 60 to 85 (P60‐85), followed by booster doses at P100 and P105. Animals were monitored and assessed weekly for body weight, NDS, grip strength, and rotarod test until they were sacrificed at P110 for further analysis. These cohorts were designed to evaluate early therapeutic efficacy and pathological outcomes through the predefined P110 endpoint, rather than long‐term disease progression or survival. Disease onset was determined by an NDS score of 1, corresponding to apparent hindlimb collapse, tremors, retraction, or slower gaits (Kim et al. 2022). NSC‐EV‐ASO treatment significantly delayed disease onset compared with vehicle or free ASOs (Figure 5B), shifting the average onset from ∼P85 in the control group to ∼P95 in the NSC‐EV‐ASO group. Although no significant differences in body weight were observed at P110 across groups (WT: 26.99 ± 0.48 g; Vehicle: 22.54 ± 0.29 g; free ASO: 22.90 ± 0.14 g; NSC‐EV‐ASO: 23.31 ± 0.29 g; Figure 5C), grip strength was significantly improved in the NSC‐EV‐ASO‐treated group (59.27 ± 2.81 N/g) compared to both the vehicle (39.38 ± 3.23 N/g) and free ASO (54.75 ± 4.00 N/g) groups (Figure 5D). Similarly, rotarod performance showed preserved motor coordination in the NSC‐EV‐ASO‐treated group (Figure 5E). Analysis of muscle mass revealed no significant differences in tibialis anterior (TA) muscle weight among groups (WT: 0.18% ± 0.02%; Vehicle: 0.16% ± 0.01%; ASO: 0.13% ± 0.01%; NSC‐EV‐ASO: 0.16% ± 0.01%; Figure 5F), but gastrocnemius (GAS) weight was significantly higher in the NSC‐EV‐ASO‐treated mice (0.48% ± 0.01%) compared with vehicle (0.40% ± 0.01%) and free ASO (0.39% ± 0.04%) groups (Figure 5G), indicating partial preservation of muscle integrity.

Because the 120‐min time point in the biodistribution assay (Figure 3C,D) primarily reflects early tissue distribution rather than long‐term CNS exposure, we next assessed the abundance of intact ASO molecules in these brain tissues at the predefined P110 using SplintR‐qPCR. This assay specifically detects full‐length ASO molecules. Quantification revealed a higher relative abundance of intact ASOs in the brains of mice treated with NSC‐EV‐ASOs compared with those receiving free ASOs (fold change: 1.39 ± 0.16, *p* = 0.008) (Figure S9). These findings indicate that NSC‐EV‐ASO‐treated mice had great brain‐associated abundance of intact ASOs at the measured endpoint following repeated dosing.

Together, these findings provide proof‐of‐concept evidence that NSC‐EV‐ASO complexes enhanced the in vivo efficacy of ASOs, likely through the combined effects of improved CNS targeting of ASOs and the intrinsic bioactivity of NSC‐EVs, resulting in delayed disease onset and improved motor function in the ALS mouse model.

### 3.6 NSC‐EV‐ASOs Reduced SOD1 Expression, Preserved Motor Neurons and Alleviated Neuroinflammation in ALS Mice

**FIGURE 6:**
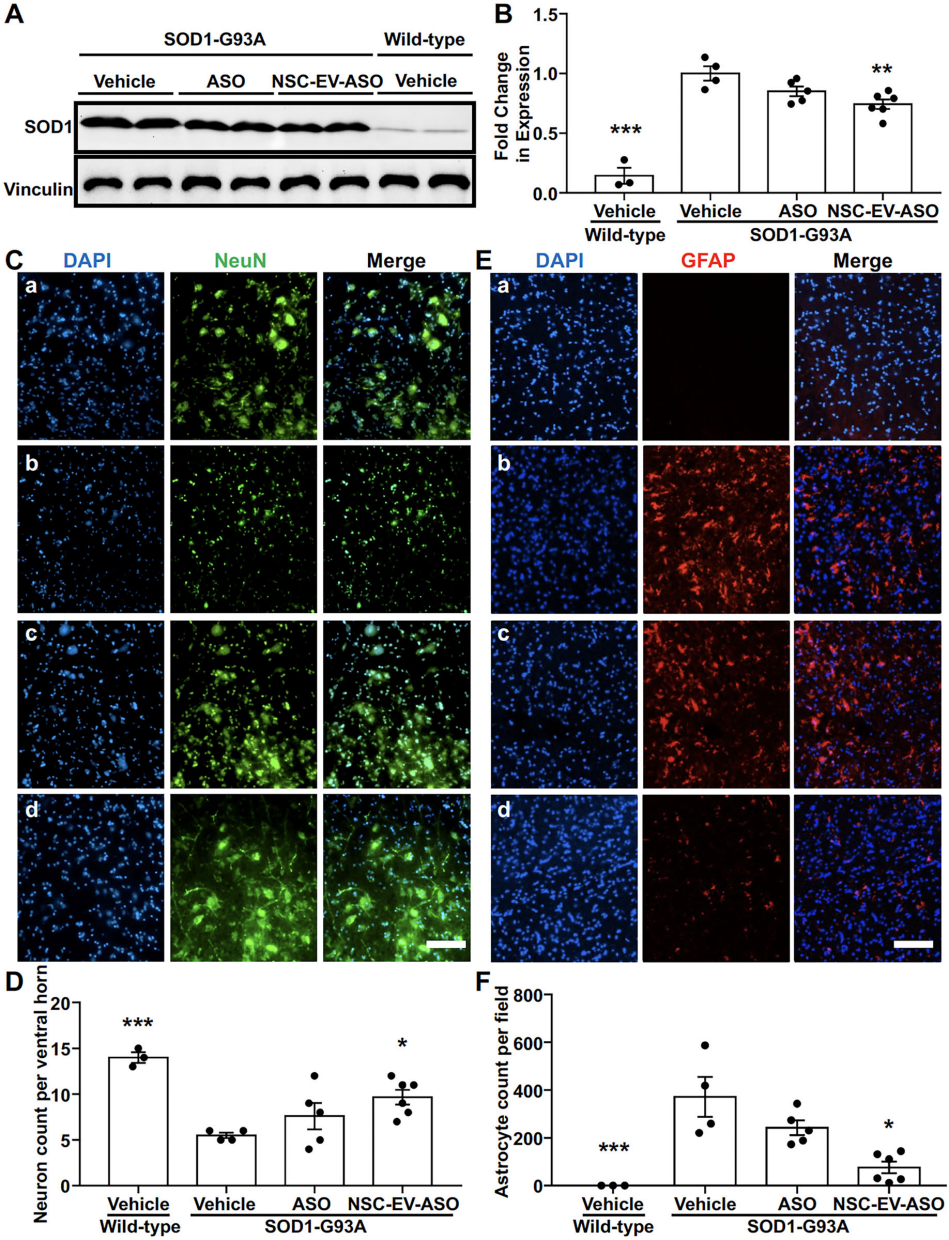
NSC‐EV‐ASOs reduced SOD1 expression, preserved motor neurons, and alleviated neuroinflammation in ALS mice. (A) Western blot analysis of SOD1 protein levels in spinal cord lysates from WT, vehicle‐treated, free ASO‐treated, and NSC‐EV‐ASO‐treated SOD1‐G93A mice. (B) Quantification of SOD1 protein levels confirms a significant reduction in the NSC‐EV‐ASO group compared to the control group. (C) Representative spinal cord immunostaining showing motor neuron survival: (a) WT, (b) vehicle, (c) free ASO, (d) NSC‐EV‐ASO. NSC‐EV‐ASO treatment markedly preserved motor neurons. Scale bar, 200 µm. (D) Quantification of motor neuron counts across groups. (E) Immunofluorescence staining of spinal cord sections for astrocyte activation marker GFAP: (a) WT, (b) vehicle, (c) free ASO, (d) NSC‐EV‐ASO. Robust astrocytic activation was observed in vehicle‐treated mice, moderately reduced by free ASOs, and markedly diminished by NSC‐EV‐ASOs. Scale bar, 200 µm. (F) Quantification of GFAP‐positive astrocyte density confirms a significant reduction in the NSC‐EV‐ASO group. Data are presented as mean ± SEM. Wild‐type (*n* = 3), vehicle‐treated (*n* = 4), ASO‐treated (*n* = 5), and NSC‐EV‐ASO‐treated (*n* = 6) mice were analyzed. Statistical significance was determined using one‐way ANOVA with Tukey's post hoc test. * *p* <0.05, ** *p* <0.01, *** *p* <0.001.

To investigate the mechanisms underlying the therapeutic effects of NSC‐EV‐ASOs, we assessed SOD1 expression, motor neuron survival and neuroinflammation in the spinal cords of SOD1‐G93A ALS mice post‐treatments. Western blot analysis revealed a significant reduction of SOD1 protein levels in NSC‐EV‐ASO‐treated mice compared to controls (Figure 6A,B).

Motor neuron degeneration and neuroinflammation are hallmark features of ALS pathology (Grad et al. 2017; Ito et al. 2016; Sances et al. 2016). Immunostaining of spinal cord sections (Figure 6C) showed extensive motor neuron loss in vehicle (b) and free ASO‐treated (c) mice compared to WT controls (a), whereas NSC‐EV‐ASO‐treated mice exhibited marked preservation of motor neurons (d). Quantification confirmed significantly higher motor neuron counts in the NSC‐EV‐ASO‐treated group (9.67 ± 0.80) compared to the free ASO‐ (7.60 ± 1.44) and vehicle‐treated (5.50 ± 0.29) groups (Figure 6D). To assess neuroinflammatory changes, we performed immunofluorescence staining for Glial Fibrillary Acidic Protein (GFAP), a marker of astrocyte activation. As shown in Figure 6E, compared to the WT group (a), vehicle‐treated ALS mice (b) showed robust astrocytic activation, which was moderately reduced by free ASOs (c) and markedly diminished in NSC‐EV‐ASO‐treated mice (d). Quantitative analysis confirmed a significant reduction in astrocyte density in the NSC‐EV‐ASO‐treated mice (76.17 ± 24.16) compared with free ASO‐ (242.00 ± 30.75) and vehicle‐treated (371.50 ± 83.67) groups (Figure 6F). Together, these results demonstrate that NSC‐EV‐delivered ASOs effectively reduce pathogenic SOD1 expression, preserve spinal cord motor neurons, and attenuate neuroinflammation, thereby contributing to delayed disease onset and improved motor function in ALS mice.

## 4 Discussion

In this study, we demonstrate that NSC‐EVs can be developed as a highly efficient, biocompatible and non‐invasive delivery platform for ASOs, enhancing CNS targeting and therapeutic efficacy in ALS. We demonstrate that NSC‐EVs efficiently load ASOs via a simple passive loading strategy without compromising vesicle integrity, significantly enhancing neuronal uptake in vitro, and achieving preferential CNS accumulation in vivo following multiple administration routes. Functionally, NSC‐EV‐ASO complexes more effectively suppress pathogenic SOD1 and C9orf72 mutant transcripts than free ASOs, delaying disease onset, mitigating neuroinflammation, and preserving motor function in the SOD1‐G93A ALS mouse model. These results establish NSC‐EVs as a clinically feasible platform that not only enhances CNS delivery of ASOs but also improves their overall therapeutic efficacy, offering a synergistic strategy to extend the therapeutic reach of RNA‐based drugs in the CNS.

ASO‐based therapies represent a promising strategy for genetic neurodegenerative diseases, exemplified by the FDA approval of Tofersen for SOD1‐linked ALS (Everett and Bucelli 2024, Miller et al. 2022). However, clinical application remains constrained by inefficient CNS penetration, limited neural targeting, and the need for repeated intrathecal or intraventricular injections that pose procedural risks and patient burden. Our findings suggest that NSC‐EVs can overcome several of these challenges. Compared to free ASOs, NSC‐EV‐associated ASOs exhibited improved stability, preferential neural cell targeting, enhanced CNS accumulation, and improved functional activity under the conditions examined. These improvements likely arise from the native composition of NSC‐EV membranes, which preserves biocompatibility and exploits endogenous trafficking mechanisms for cellular communication and BBB transport. Mechanistically, the superior delivery efficiency of NSC‐EVs may stem from both their cellular origin and intrinsic membrane features (Edelmann and Kima 2022; Hallal et al. 2022; Liam‐Or et al. 2024). NSCs secrete EVs enriched in adhesion and guidance molecules such as Neural Cell Adhesion Molecule 1 (NCAM1), L1 Cell Adhesion Molecule (L1CAM), and integrins, which have been implicated in neuron‐glia communication and CNS tropism (Chiu et al. 2025, Collier et al. 2025; Gomes and Witwer 2022; You et al. 2022). These surface components likely promote receptor‐mediated endocytosis or membrane fusion with neural cells, thereby contributing to the uptake of EVs and their translocation across the BBB, although their specific roles in the delivery properties remain to be determined. In addition, the lipid raft‐rich membranes of NSC‐EVs likely protect ASOs from extracellular nucleases and reduce rapid renal clearance (Zhang et al. 2025), collectively improving bioavailability and CNS targeting. Consistently, our comparison with HEK 293T‐derived EVs suggest that EV cellular origin may contribute to differences in brain biodistribution under the conditions examined, although future studies are warranted to define the molecular basis of these differences.

The evaluation of multiple administration routes in this study further highlights the translational versatility of the NSC‐EVs. Intracerebroventricular (*i.c.v*.) injection provided robust CNS exposure but remains invasive. In contrast, intranasal (*i.n*.) administration achieved substantial brain distribution without surgical intervention, likely through dual mechanisms: systemic absorption followed by BBB permeability and direct transport via olfactory and trigeminal pathways, thus bypassing the BBB (Erdő et al. 2018). Although the specific transport mechanisms were not investigated in this study, our findings support the potential of this route for chronic neurodegenerative diseases (Dagda et al. 2022; Lv et al. 2023), where repeated and non‐invasive dosing may be advantageous. Meanwhile, systemic (*i.v*.) administration yielded broad CNS and peripheral distribution under the tested conditions, suggesting potential applications in disorders with both central and systemic pathology. Although practical considerations such as dosing efficiency, variability in nasal absorption, and cross‐species scalability remain important challenges for clinical translation, our findings demonstrate that NSC‐EVs can preserve ASO integrity and enable targeted delivery across diverse routes, supporting their potential adaptability for translational use.

Therapeutically, NSC‐EV‐ASO treatment slowed disease progression and improved motor function in SOD1‐G93A ALS mice, accompanied by preservation of spinal motor neurons and reduced neuroinflammation. These effects correlated with reduced pathogenic SOD1 expression, confirming a direct pharmacological mechanism. Importantly, the observed therapeutic benefits likely result from the synergistic action of both the ASO cargo and the intrinsic bioactivity of NSC‐EVs (Narayanappa et al. 2025). Prior studies have shown that EVs derived from neural or stem cell sources can attenuate microglial activation (Tian et al. 2021, You et al. 2022), reduce oxidative stress (Zhao et al. 2025), and support neuronal repair (Gu et al. 2023). Endogenous NSC‐EV cargos, including neuroprotective miRNAs and proteins, may complement ASO‐mediated gene regulation, thereby providing additional therapeutic effects (Mohammed et al. 2020; Zhang et al. 2021). This dual‐action mechanism distinguishes NSC‐EVs from synthetic delivery systems, where carriers are typically inert. Beyond ALS, our findings hold broader implications for nucleotide acid‐based therapeutics targeting the CNS. The demonstrated delivery efficiency of NSC‐EVs across multiple routes may apply to other oligonucleotide modalities, including siRNAs, miRNA mimics and CRISPR‐based genome editors (Liu et al. 2023). Their inherent neural tropism positions NSC‐EVs as versatile shuttles for nucleic acid delivery in neurological disorders such as Huntington's disease (Imbert et al. 2019), frontotemporal dementia (Tran et al. 2022), or Parkinson's disease (Cole et al. 2021).

At the reduced cumulative dose tested (∼28 µg ASO per mouse), we observed a ∼10‐day delay in disease onset. Although modest, this effect is notable given the substantially lower ASO exposure compared with doses commonly used in previous studies in the ALS mouse model (∼350 µg) (Jiang et al. 2016). In addition to delayed disease onset, NSC‐EV‐ASO‐treated mice showed improved motor performance, including enhanced rotarod performance and grip strength, further supporting the functional benefits of this therapeutic strategy. However, a comprehensive evaluation of disease progression, particularly survival to end‐stage, was not performed, as this study was primarily a proof‐of‐concept investigation focused on delivery efficiency and therapeutic feasibility. Future studies with larger cohorts, optimized dosing regimens and appropriately powered longitudinal analyses will be required to more rigorously assess overall disease progression, survival benefit and the durability of therapeutic effects.

It should be noted that NSC‐EV‐ASO preparations used for both in vitro and in vivo functional evaluations were not subjected to nuclease pre‐treatment prior to administration. Therefore, we cannot exclude the possibility that a fraction of surface‐associated ASOs contributed to the observed functional effects. Although nuclease protection assays indicate that a substantial portion of ASOs is protected from nuclease degradation after association with EVs, these experiments do not distinguish between luminally encapsulated and surface‐bound ASOs. Clarifying the relative contributions of different loading states will require further investigation. Surface‐associated and luminally localized ASOs may exhibit distinct intracellular delivery behaviours (Bost et al. 2021; Didiot et al. 2016; Kapustin et al. 2021). Surface‐associated ASOs may be more readily exposed to the extracellular or intracellular environment following EV‐cell interactions, potentially allowing earlier release or target engagement. In contrast, luminally localized ASOs would be expected to remain protected by the EV membrane during extracellular transport but would require release from the EV membrane and subsequent intracellular trafficking before engaging target transcripts. These differences could influence the kinetics of ASO release, intracellular trafficking and target engagement. In the present study, LysoTracker analysis showed that EV‐associated ASO fluorescence was initially associated with lysosomal compartments and decreased over time, however, these observations do not distinguish the behaviour of surface‐associated versus luminally localized ASOs or directly establish endosomal escape. The relative contribution of these loading states to cellular uptake, intracellular trafficking, ASO release and functional gene silencing remains unknown and warrant dedicated mechanistic investigations.

We also observed differential effects across skeletal muscle groups, with increased gastrocnemius weight but no significant change in tibialis anterior. Such variability is consistent with previous findings that skeletal muscles exhibit heterogeneous patterns of denervation and degeneration, and that individual muscles may respond differently to therapeutic interventions (Harrison and Rafuse 2020; Smittkamp et al. 2014). Differences in fibre‐type composition (Giacomello et al. 2020; Santocildes et al. 2022), along with variations in disease stage (Smittkamp et al. 2014) and local neuromuscular vulnerability (Harrison and Rafuse 2020), may influence the extent of muscle‐specific responses to treatment. Future studies incorporating comprehensive muscle analyses and quantitative biodistribution measurements will help clarify tissue‐specific therapeutic responses.

Compared with synthetic carriers such as lipid nanoparticles or polymeric vesicles (Byrnes et al. 2023; Shirley et al. 2020), NSC‐EVs offer intrinsic biocompatibility, low immunogenicity and the ability to engage physiological transport pathways. They also demonstrated superior stability and protection of nucleic acid cargo, leading to prolonged therapeutic effects (Didiot et al. 2016). Nonetheless, synthetic systems retain advantages in terms of large‐scale reproducibility and manufacturing control. Integrating EV biology with synthetic nanotechnology, for example, by surface engineering or hybrid vesicle design, may further enhance delivery precision and scalability (Jang et al. 2013; Tian et al. 2014; Yu et al. 2021).

While promising therapeutic efficacy of NSC‐EV‐delivered ASOs was observed in the ALS mouse model, several limitations warrant consideration. First, the molecular mechanisms underlying EV‐ASO loading, cellular uptake and endosomal escape remain poorly understood. Whether ASOs are primarily encapsulated within the EV lumens or associated with their surface could affect release dynamics and pharmacokinetics (Kuriyama et al. 2021). In this study, LysoTracker‐based imaging revealed that a large proportion of NSC‐EV‐ASO‐associated fluorescence signals initially co‐localized with lysosomal compartments, indicating trafficking through the endo‐lysosomal pathway, followed by a gradual reduction in co‐localization over time. This observation is consistent with previous reports suggesting that EVs may facilitate endosomal escape through mechanisms such as membrane fusion (Bebelman et al. 2020; Bonsergent et al. 2021; Liu et al. 2023), lipid‐mediated destabilization of endosomal membranes (Heath et al. 2019; Shete et al. 2014), or involvement of EV‐associated proteins that regulate intracellular trafficking (Brock et al. 2018; Gandek et al. 2023). However, the extent to which NSC‐EVs influence endosomal escape requires further investigation, using orthogonal approaches such as subcellular fractionation, radiolabel tracing, or established endosomal escape reporter systems. Second, biodistribution analysis in this study relied primarily on fluorescence labelling (Atto‐647), which does not distinguish intact ASOs and degraded fragments. More quantitative approaches, such as PCR‐based detection or radiolabelling (Busato et al. 2016), will be needed to more precisely define CNS exposure and clearance of ASOs delivered by NSC‐EVs. Although our data (Figure S9) indicate that NSC‐EV‐mediated delivery enhances the accumulation of intact ASOs in the brain and is accompanied by improved molecular and functional outcomes in the ALS mouse model, a direct causal relationship between CNS accumulation and therapeutic efficacy cannot be fully established from measurements obtained at a single time point. Longitudinal pharmacokinetic studies will be important for clarifying ASO retention and clearance dynamics in the CNS. In addition, although SplintR‐qPCR provides evidence for increased intact ASO abundance following NSC‐EV‐ASO administration, the current assay was used for relative comparison rather than absolute quantification of ASO concentration in brain tissue. Factors including tissue recovery efficiency, assay background and normalization strategy may influence quantitative interpretation. Future studies incorporating validated standard curves, absolute quantification approaches and multi‐timepoint pharmacokinetic analyses will be important to precisely define CNS exposure, retention and clearance of EV‐delivered ASOs.

Finally, further studies will be required to comprehensively evaluate the translational potential of this platform. In particular, therapeutic assessments should include optimized dosing paradigms, long‐term safety, immunogenicity and durability in longitudinal studies. Comprehensive safety evaluation should also incorporate global transcriptomic analyses to assess potential ASO‐related off‐target effects, as well as systemic characterization of innate immune activation, inflammatory gene induction and immune responses to EV‐associated components. In addition, peripheral accumulation following systemic administration and potential off‐target effects in non‐CNS tissues should be carefully monitored.Standardization of NSC‐EV production will also be essential for clinical development, including controlled cell culture conditions, improved EV isolation and purification methods and rigorous quality control criteria to minimize batch‐to‐batch variability and ensure reproducibility and scalability of EV manufacturing. In addition, the current study focused on the optimized loading condition identified in our experiments (25k ASO/EV), while higher loading ratios were not systematically evaluated for cellular compatibility or intracellular trafficking. Future studies will be needed to determine how increasing ASO loading influences EV integrity, cellular viability, intracellular trafficking and delivery efficiency, thereby defining an appropriate loading range for further development of the platform.

Despite these challenges, our study advances the concept of using endogenous EVs as precision delivery vehicles for RNA therapeutics (Bader et al. 2025; Bader et al. 2024). By leveraging the natural tropism and molecular machinery of NSCs, NSC‐EVs achieve efficient and targeted delivery of ASOs to the CNS, overcoming longstanding barriers to gene‐targeted therapy. This biologically inspired strategy aligns with emerging trends in regenerative nanomedicine, in which both the therapeutic cargo and its carrier contribute to efficacy. Future studies integrating proteomic and lipidomic profiling may help identify surface molecules responsible for CNS targeting, guiding rational engineering of EV tropism. The modularity of NSC‐EVs enables co‐loading of therapeutic and diagnostic cargos (‘theragnostic’ EVs) for real‐time monitoring and adaptive dosing. Combining NSC‐EV therapy with neuroprotective agents or gene‐editing interventions may yield synergistic benefits in multifactorial neurodegenerative diseases (Lawson et al. 2022; Madhu et al. 2024; Zheng et al. 2021). Ultimately, studies in large animal and humanized models are essential for bridging the translational gap and evaluating clinical dosing, manufacturability and safety.

In conclusion, our study establishes NSC‐EVs as a natural, scalable and clinically adaptable platform for targeted nucleic acid delivery to the CNS. By improving ASO stability, distribution, and efficacy, NSC‐EVs help overcome major barriers to nucleic acid‐based interventions for genetic and degenerative brain disorders. These findings extend the therapeutic utility of EVs and highlight the potential of harnessing regenerative cell‐derived nanovesicles as next‐generation delivery systems for precision and efficient gene therapy in the human CNS.

## Author Contributions

**Yaochao Zheng**: conceptualization, data curation, formal analysis, investigation, methodology, project administration, writing – original draft, writing – review and editing. **Taylor Jade Ellison**: conceptualization, data curation, investigation, methodology, project administration, writing – original draft, writing – review and editing, formal analysis. **Robert Garrard**: data curation. **Jinghui Gao**: data curation. **Xinning Lai**: data curation. **Lin Lan**: data curation. **Jin Xie**: writing – review and editing. **Jie Jiang**: conceptualization, funding acquisition, writing – review and editing. **Yao Yao**: conceptualization, funding acquisition, project administration, writing – original draft, writing – review and editing.

## Conflicts of Interest

The authors declare that they have no known competing financial interests or personal relationships that could have influenced the work reported in this paper.

## Supporting information




**Supporting Information**: jev270370‐sup‐0001‐FigureS1‐S9.docx

## Data Availability

The data that support the findings of this study are available from the corresponding author upon reasonable request.
