# Supplementary material for: Neural Stem Cell‐Derived Extracellular Vesicles Improve CNS Delivery and Therapeutic Efficacy of Antisense Oligonucleotide in Amyotrophic Lateral Sclerosis
Source: J Extracell Vesicles. 2026 Sep 15;15(9):e70370. doi: 10.1002/jev2.70370 (PMC13576945; doi:10.1002/jev2.70370)
Supplement: Supplementary file 1 [file JEV2-15-e70370-s001.docx]

**Supplementary Figures and Figure Legends**

**Supplementary Figure S1.** (A) Western blot analysis of canonical EV markers (Alix, Flotillin-1, Syntenin-1, and CD9) and the intracellular negative marker Calnexin in NSC-EVs and ASO-associated NSC-EVs (NSC-EV-ASO). NSC cell lysate was included as a control. (B) Nanoparticle tracking analysis (NTA) of NSC-EVs before and after ASO loading. (C) Comparison of mean NSC-EV diameters from NTA size distribution analysis, showing no significant change after ASO loading. Statistical significance was determined using one-way ANOVA with Tukey’s post hoc test. n.s., not significant.

**Supplementary Figure S2. Schematic representation of the therapeutic ASO design.** The ASO was synthesized with a phosphorothioate backbone and 2′-O-methoxyethyl (2′-MOE) modifications, and conjugated at the 3′ end with an Atto-647N fluorophore for visualization.

**Supplementary Figure S3.** **DNase protection assay demonstrates protection of ASOs associated with NSC-EVs.** NSC-EV-ASOs were treated with DNase in the absence or presence of detergent (SDS), and the preparations were subsequently quantified by SplintR-qPCR. Free ASO samples were included as controls. DNase treatment completely abolished the signal from free ASO samples, confirming efficient nuclease digestion. In contrast, a substantial fraction of the EV-associated ASO signal remained detectable after DNase treatment, indicating protection from nuclease degradation. This protective effect was lost when NSC-EV-ASO samples were pre-treated with detergent prior to DNase digestion. Each dot represents a biological replicate, which consists of three technical replicates. Statistical significance was determined using one-way ANOVA with Tukey’s post hoc test. * *p* <0.05, ** *p* <0.01, *** *p* <0.001. n.s., not significant.

**Supplementary Figure S4. Lysosomal localization of NSC-EV-delivered ASOs in NSC-34 cells.** NSC-34 Cells were stained with LysoTracker and then treated with NSC-EV-associated fluorescently labeled ASOs. Colocalization between NSC-EV-ASOs and LysoTracker-positive compartments was quantified by confocal microscopy to assess lysosomal trafficking and potential lysosomal escape of NSC-EV-delivered ASOs. Each dot represents a biological replicate, which consists of three technical replicates. Statistical significance was determined using one-way ANOVA with Tukey’s post hoc test (each time point compared with 70 mins). * *p* <0.05, ** *p* <0.01, *** *p* <0.001. n.s. (not significant) is not shown.

**Supplementary Figure S5.** **Enhanced brain accumulation of ASOs delivered by NSC-EVs compared to non-neural EVs following intravenous administration.** NSC-EVs or HEK293T cell-derived EVs (293T-EVs) were loaded with fluorescently labeled ASOs (293T-EV-ASOs) using the same loading condition (25K ASO/EV). Following ASO loading, EV particle numbers were quantified by nanoflow cytometry and normalized to equivalent particle input prior to intravenous administration. Brain tissues were collected and imaged *ex vivo* using an in vivo imaging system (IVIS) to assess ASO distribution. Representative IVIS images are shown. Mice treated with NSC-EV-ASOs exhibited higher ASO signal intensity in brain tissue compared with those treated with 293T-EV-ASOs. N = 3 in each group were analyzed.

**Supplementary Figure S6**. Representative fluorescence images show robust uptake and intracellular retention of fluorescently labeled ASOs in SOD1-GFP-overexpressing NSC-34 cells after 48 h treatment with NSC-EV-ASOs. The ASOs exhibit an exclusive, non-colocalizing pattern relative to SOD1-GFP, occupying distinct intracellular compartments.

**Supplementary Figure S7.** **Comparison of ASOs delivered by NSC-EVs or liposomes on the viability of NSC-34 cells overexpressing mutant SOD1.** NSC-34 cells overexpressing ALS-associated mutant SOD1 (SOD1^G93A^) were treated with NSC-EVs alone, liposomes alone, liposome-loaded ASOs (lipo-ASO), NSC-EV-ASOs, or vehicle control under comparable dosing conditions normalized to particle number and equivalent ASO dose. Cell viability was assessed using the CCK-8 assay. Each dot represents a biological replicate, which consists of three technical replicates. Statistical significance was determined using one-way ANOVA with Tukey’s post hoc test. * *p* <0.05, ** *p* <0.01, *** *p* <0.001. n.s., not significant.

**Supplementary Figure S8**. **NSC-EV-ASO-C9 selectively reduces repeat-containing transcripts and RNA foci in C9-HRE ALS fibroblasts**. **(A)** RT-qPCR analysis shows selective reduction of C9-HRE transcripts after NSC-EV-ASO-C9 treatment compared to free ASOs at equivalent doses. **(B)** Independent RT-qPCR with primers spanning exon 2 confirmed that overall C9orf72 expression was not affected, indicating selective depletion of repeat-containing transcripts. **(C-D)** RNA fluorescence in situ hybridization (FISH) images and quantification show a significant reduction in nuclear RNA foci in patient-derived fibroblasts treated with NSC-EV-ASO-C9, whereas free ASOs were notably less effective. Each dot represents a biological replicate, which consists of three technical replicates. Statistical significance was determined using one-way ANOVA with Tukey’s post hoc test. Scale bar, 10 μm. * *p* <0.05, ** *p* <0.01, *** *p* <0.001.

**Supplementary Figure S9.** **Detection of full-length ASOs in the mouse brain following intranasal administration.** Brain tissues from ALS mice treated with free ASOs, NSC-EV-ASOs, or vehicle were collected at the predefined P110 endpoint following repeated intranasal administration (as shown in Fig.5A), and analyzed using SplintR-qPCR, which is designed to detect the intact ASO molecules. Data are presented as mean ± SEM, with the vehicle group used as the baseline reference for relative comparison. Vehicle-treated (n = 4), ASO-treated (n = 5), and NSC-EV-ASO-treated (n = 6) mice were analyzed. Statistical significance was determined using one-way ANOVA with Tukey’s post hoc test. * *p* <0.05, ** *p* <0.01, *** *p* <0.001.
